# Weakly supervised text classification on free-text comments in patient-reported outcome measures

**DOI:** 10.3389/fdgth.2025.1345360

**Published:** 2025-04-30

**Authors:** Anna-Grace Linton, Vania Gatseva Dimitrova, Amy Downing, Richard Wagland, Adam W. Glaser

**Affiliations:** ^1^ UKRI CDT in AI for Medical Diagnosis and Care, University of Leeds, Leeds, United Kingdom; ^2^ School of Computing, University of Leeds, Leeds, United Kingdom; ^3^ School of Medicine, University of Leeds, Leeds, United Kingdom; ^4^ School of Health Sciences, University of Southampton, Southampton, United Kingdom; ^5^ Leeds Institute of Medical Research, University of Leeds, Leeds, United Kingdom

**Keywords:** free-text, text classification, patient-reported data, short text, weakly supervised, natural language processing, PROMS, patient-generated data

## Abstract

**Background:**

Free-text comments in patient-reported outcome measures (PROMs) data provide insights into health-related quality of life (HRQoL). However, these comments are typically analysed using manual methods, such as content analysis, which is labour-intensive and time-consuming. Machine learning analysis methods are largely unsupervised, necessitating post-analysis interpretation. Weakly supervised text classification (WSTC) can be a valuable analytical method of analysis for classifying domain-specific text data, especially when limited labelled data are available. In this paper, we applied five WSTC techniques to PROMs comment data to explore the extent to which they can be used to identify HRQoL themes reported by patients with prostate and colorectal cancer.

**Methods:**

The main HRQoL themes and associated keywords were identified from a scoping review. They were used to classify PROMs comments with these themes from two national PROMs datasets: colorectal cancer (*n* = 5,634) and prostate cancer (*n* = 59,768). Classification was done using five keyword-based WSTC methods (anchored CorEx, BERTopic, Guided LDA, WeSTClass, and X-Class). To evaluate these methods, we assessed the overall performance of the methods and by theme. Domain experts reviewed the interpretability of the methods using the keywords extracted from the methods during training.

**Results:**

Based on the 12 papers identified in the scoping review, we determined six main themes and corresponding keywords to label PROMs comments using WSTC methods. These themes were: Comorbidities, Daily Life, Health Pathways and Services, Physical Function, Psychological and Emotional Function, and Social Function. The performance of the methods varied across themes and between the datasets. While the best-performing model for both datasets, CorEx, attained weighted F1 scores of 0.57 (colorectal cancer) and 0.61 (prostate cancer), methods achieved an F1 score of up to 0.92 (Social Function) on individual themes. By evaluating the keywords extracted from the trained models, we saw that the methods that can utilise expert-driven seed terms and extrapolate based on limited data performed the best.

**Conclusions:**

Overall, evaluating these WSTC methods provided insight into their applicability for analysing PROMs comments. Evaluating the classification performance illustrated the potential and limitations of keyword-based WSTC in labelling PROMs comments when labelled data are limited.

## 1 Introduction

Patients’ perspectives on their health have become increasingly important when assessing the quality of survival among individuals diagnosed with cancer. These perspectives are considered key for a more holistic interpretation and understanding of their health conditions and health-related quality of life (HRQoL) (1, 2). Patient-reported outcome measures (PROMs) provide a value assessment of a patient’s HRQoL through a combination of close-ended questions, such as Likert scales, and open-ended questions (3). The free-text comments received in response to the open-ended questions in PROMs are brief but can provide additional details that complement the closed questions. This additional information allows for a more holistic understanding of the nuances and factors influencing the patient’s health status (4, 5).

Although responses to close-ended questions in PROMs can be analysed efficiently using statistical methods, analysing free-text responses presents challenges. Consequently, such data are often left unexplored in clinical research (6–9). The analysis of PROMs comments is significantly more time- and resource-demanding than the processing of closed-question responses. This task is typically conducted manually using qualitative analysis methods, which are susceptible to subjectivity and lack scalability. Moreover, the analyses are data-dependent, with variability in topics extracted from the data, limiting comparison between datasets collected from different cohorts and populations. Often, the analysis of the comments is omitted from the analysis of PROMs data, which can result in a loss of information and potential bias in the reported findings (10, 11). The demands of analysis are intensified by the increased use of patient-reported data, such as PROMIS and other PRO initiatives (12), and patient experience surveys collecting thousands of free-text responses each year, which would take months to go through manual review (13). The time required to analyse these comments can exceed the usefulness of the insights they contain.

Automated analysis of free-text comments in PROMs can be enabled through the adoption of text analytics methods, but it poses key challenges. Principally, PROMs free-text data are usually unlabelled. These data come from patients in a free format and can relate to anything that patients want to raise about their quality of life. Unsupervised classification methods, which are often adopted in practical applications, offer solutions where topics and insights are derived from the specific datasets used. Therefore, the derived findings and topics depend on the specific datasets and do not generalise to other datasets (14). A further challenge in finding appropriate text analytics methods to analyse PROMs comments is the size of the data. The individual contributions are typically brief. For example, the Living with and Beyond Bowel Cancer datasets (15) had a mean of 43 words per comment. In addition, the datasets are often not large enough for machine learning methods, as free-text comments are optional in PROMs surveys, and not all patients provide such responses.

One approach to addressing these challenges is to adopt weakly supervised text classification (WSTC). WSTC is increasingly used when there is insufficient labelled data or it is costly to obtain expert annotations (16, 17). Instead of relying on labelled data, WSTC uses weak supervision signals during training, such as keywords or heuristics, to classify text (18). Consequently, the need for a large, annotated corpus can be avoided, which makes the approach quite appealing for analysing PROMs comment data. Furthermore, keyword-based WSTC can allow guidance from domain experts and thus can build on healthcare research related to patients’ quality of life. Although WSTC shows promise, its performance on PROMs comments is uncertain, as does its suitability for adoption in this and broader healthcare contexts. Furthermore, for WSTC to be effectively used to classify PROMs comments, a reliable set of HRQoL themes is needed.

In this paper, we investigated the extent to which WSTC can be adopted to enable automatic classification of patients’ free-text comments in PROMs data. We explored this in the context of free-text comments collected through NHS PROMs surveys as part of a PhD project aimed at examining the value of PROMs comments.

This paper presents a framework for using WSTC to classify free-text comments in PROMs datasets. First, key themes and corresponding keywords related to HRQoL in free-text comments were identified based on a scoping review reported by Linton (19). Second, five keyword-based WSTC methods, namely, BERTopic (20), CorEx Algorithm (CorEx) (21), Guided LDA (GLDA) (22), WeSTClass (23), and X-Class (24) were applied to label free-text data from two PROMs surveys with the predefined key themes using seed terms. The performance of the algorithms was analysed, and the insights were presented to the clinical research team to discuss the feasibility of using WSTC for PROMs comment classification.

## 2 Relevant work

### 2.1 Analysing free-text comments in PROMs

Studies analysing free-text in patient-reported text data (including PROMs and patient-reported experience data) have employed both supervised and unsupervised approaches (14). Most automated approaches to analyse patients’ free-text data rely on unsupervised techniques using information extraction (25) and classification (7, 9, 26, 27).

Spasic et al. (28) mapped free-text comments from knee osteoarthritis patients to the Likert scales of a PROMs dataset by performing sentiment analysis and using MetaMap (29) to look up a lexicon for named entity recognition. To analyse free-text comments from an Irish in-patient survey, Robin et al. (30) used Saffron software to extract key terms in the medical domain and automatically mapped them to predefined categories. In these studies, the authors standardised and grouped responses using information extraction approaches to reduce manual effort in analysis. A significant amount of manual effort was required to provide annotated data to validate keyword extraction methods. While these methods allow a keyword-level analysis, they do not extend to thematic grouping.

Several studies have used unsupervised classification methods to derive the main themes in a corpus of free-text comments. Wagland et al. (7) utilised unsupervised machine learning algorithms to identify the main themes of patient experiences, which allowed them to see the impact of care on health-related quality of life, which was verified using qualitative analysis. Similarly, Arditi et al. (9) used text classification to derive the main themes in free-text comments from the Swiss cancer Patient Experience Survey. The derived themes were related to personal and emotional experiences and consequences of living with cancer and receiving care. Along the same line of research, Pateman et al. (26) utilised a text analytics tool to identify the main themes in patients’ free-text comments about their experiences and quality-of-life outcomes in head and neck cancers. They extracted a concept map that identified main keyword clusters and linked them based on common terms. However, these methods are largely limited by the resources and domain expertise needed to interpret the themes and the relevance of the derived themes.

Recent studies have employed mixed-method approaches to analyse and evaluate large patient-reported text datasets, thereby providing an assessment of usefulness and insights into automatic thematic extraction. Sanders et al. combined text analytics and manual qualitative analysis to explore the usefulness of patient experience data in services for long-term conditions (31). They discovered that comments gave meaning to otherwise meaningless quantitative scores, such as “neither likely nor unlikely,” and polarised scores, such as strongly disagree/strongly agree. The authors argued that digital collection and automated analysis produced broad topics, but, compared to qualitative analysis, were more time- and resource-efficient. These methods show that grouping comments in themes is helpful for healthcare research. However, the findings from these methods are data-dependent. Crucially, they still require additional human effort to analyse the themes and put meaningful labels, which can introduce subjectivity.

In our paper, we propose a weakly supervised approach to identify the main themes in a corpus of patient comments. Rivas et al. (13) used a supervised approach to develop a tool for automatically conducting thematic analysis on a Welsh cancer patient experience survey to identify themes. A rule-based information extraction was used and developed through co-design with healthcare researchers. The approach has the benefit of being able to be systematically applied to patient experience data to summarise the data. However, rule extraction approach required significant effort and the themes were defined based on the dataset used during development, making it hard to transfer to another dataset without significant effort. Similar to Rivas et al. (13), our proposed framework aims to classify PROMs comments into predefined themes. In contrast, our proposed framework leverages generalised themes derived from a scoping review of qualitative research that has analysed PROMs comments. By employing weakly supervised short-text classification methods, we aim to classify patients’ free-text comments into these predefined themes, providing a more versatile and transferable solution.

### 2.2 Weakly supervised short text classification

Short text classification has gained significant attention with the increase in generated short texts, such as social media posts, presenting challenges like ambiguity and data sparsity, which makes information extraction difficult (32, 33). Short text classification focuses on overcoming the challenges of classifying short texts such as inadequate length and low word frequency, which often lead to ambiguity due to lack of contextual information (32, 34–36). Some methods attempt to enrich the contextual information of short text using external information from knowledge bases (34, 37). However, this requires the existence or creation of knowledge bases for that domain, which require expertise and can be time-consuming. In addition, many short text classification methods, in particular, deep neural network approaches, require a large amount of annotated data, which, as described previously, is often not possible or readily available when dealing with patients’ free-text comments, resulting in a barrier to frequent application.

WSTC uses weakly supervised signals for text classification and overcomes the challenge of small amounts of labelled data (18). For classification, it employs signals such as labelled documents (38, 39), keywords representative of the class (24, 40–43), or heuristic rules (16, 44, 45). These methods make it possible to automatically create training data rather than labelling data by hand, alleviating the bottleneck associated with the need for labelled data.

Keyword- or seed term-based WSTC has proven to be a popular approach, as it allows users to provide a set of keywords for each class, providing pseudo-labels or weak signals of the class. The set of keywords can be extensive or very short (43, 46). Meng et al. (23) used seed terms or class labels provided by the user as weak supervision to generate pseudo-documents to pre-train a neural classifier, which is then refined through a self-training module with bootstrapping. Mekala and Shang (42) used contextualised representations of a few human-provided seed words for pseudo-labelling of a contextualised corpus on two real-world long text datasets. Gallagher et al. (21) used user-provided seed terms to incorporate domain knowledge into CorEx to enable the guiding and interpretation of topics with “minimal human intervention.” Importantly, these methods require keywords suitable to the text being classified. Therefore, a reliable set of keywords is required for PROMs comment analysis.

Keyword-based WSTC methods have been applied to user-generated text datasets, but these datasets tend to be more curated or significantly larger than available PROMs datasets. For example, SentiHood (47), a SemEval dataset, has been evaluated for distinct categories, with general/miscellaneous text grouped and irrelevant and uncertain comments categorised as such and removed. Similarly, Yelp Review (48) contains two categories (good/bad) and over 1 million samples of text. Likewise, in the New York Times Annotated Corpus (49), each document had a single ground truth label such as business, sports, and politics and at least 100 instances of each topic. While WSTC shows promising results on various user-generated corpora, to the best of our knowledge, it has not been applied to patient-reported text data.

Based on the highlighted gaps, the work presented in this paper aims to investigate the extent to which WSTC can be adopted for free-text comments in PROMs. We focus on keyword-based classification for short text as it allows domain experts to directly contribute their domain knowledge in a cost-effective manner, which is advantageous for the adoption of an analytical method for PROMs and other healthcare-related texts. We critically analyse the feasibility of using WSTC for classifying PROMs comments.

## 3 Framework for PROMs comment classification

**Figure 1:**
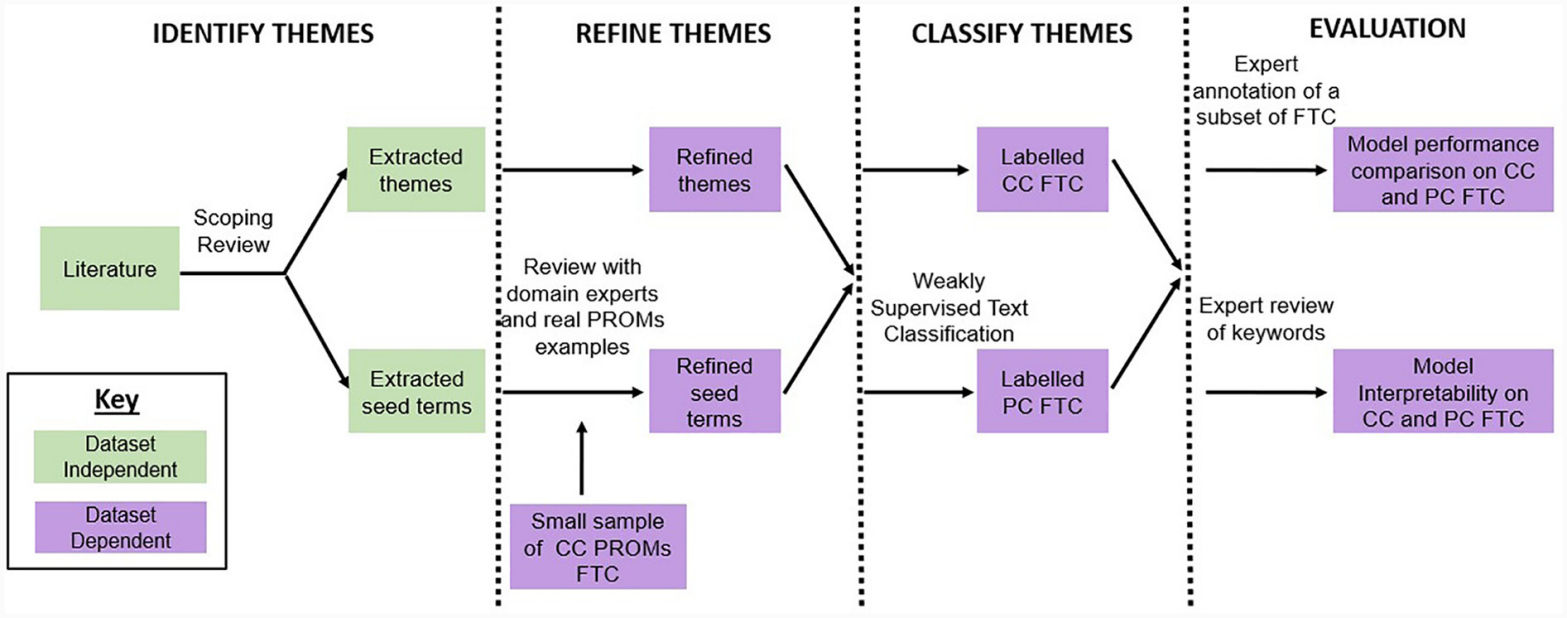
Framework for the automated analysis of free-text comments in PROMs. The themes to identify in the PROMs comments are selected using themes found in a scoping review and refined by domain experts. The performance of five keyword-based WSTC methods is evaluated on colorectal cancer (CC) and prostate cancer (PC) PROMs comment datasets.

The main aim of our work is to develop a generic approach for analysing free-text comments that can be adopted by health researchers to gain deeper insights into PROMs data. We propose a framework that can be applied to free-text comments in any PROM questionnaire. The framework consists of four main steps: (1) identifying themes of patient-reported HRQoL, gathered through a scoping review; (2) refining these themes using real-world examples; (3) using these themes to classify comments using WSTC on two real-world PROMs datasets; and (4) evaluating the models based on the quantitative performance and human-interpretable outputs of the methods. Figure 1 describes the overall framework, with each step described in the following sections.

## 4 Identifying themes

### 4.1 Scoping review: literature search and study selection

We conducted a scoping review to determine themes related to HRQoL commonly reported by patients with chronic conditions in PROMs comments. Based on Munn et al. (50), who compared the purpose of various types of reviews, a scoping review was selected to identify emerging themes and systematically map them to extract the themes relevant to the classification of PROMs comments. Through further refinement of the themes, we aimed to identify a set of reliable themes to serve as classification labels for PROMs comments. Therefore, the review aimed to answer, “What are the themes of HRQoL and QoL commonly reported by patients with chronic illnesses in free-text comments of patient-reported outcome data?” [full details of the scoping review have been reported in Chapter 4 in the study by Linton (19)].

The studies were screened in two stages: an initial review of titles and abstracts, followed by a full-text review. One author (A-GL) independently performed the literature search, eligibility assessments, and study selection, which was then reviewed by the other authors. Studies were retrieved by searching the following databases: Medline via OvidSP, Embase via OvidSP, and PubMed. We derived the search terms from the main concepts in the search question, such as “patient-reported outcome,” “patient-reported experience,” “free-text,” and “chronic illness.” We focussed on studies on patient-reported outcomes only but included patient-reported experiences due to inconsistency in the reporting language. The study selection included an analysis of free-text comments only, excluding themes from transcripts or patient narratives. We retrieved studies published between 2011 and 2021. The retrieved publications were deduplicated using the Zotero reference manager. This set of papers was given to the experts to determine the suitability and whether any relevant studies were missing. For the selected studies, metadata, such as publication year and patient group were collected and recorded and the MMAT appraisal tool (51) was used to appraise studies.

### 4.2 Data extraction and synthesis

Topics from the selected studies were extracted, tabulated, and grouped into main themes. From each study, we extracted titles, authors, publication year, country, patient group (disease type), size of the data, length of documents in the dataset, and methods used. The topics selected were those mentioned in the free-text comments based on the patients and recorded by the authors of the selected studies. The extracted topics were grouped by semantic similarity. Topics with a prevalence greater than seven were chosen as the main themes for identifying in the PROMs comments. We describe themes as a group of related topics. The themes were reviewed and validated by three domain experts (AD, AWG, RW), who also clarified their definition and regrouped them to better align with the WHO Quality of Life (WHOQoL) framework for improved understanding.

For each theme, associated terms were captured to be used as seed terms for annotation and by the keyword-based WSTC models. Seed terms are words or phrases representative of each theme. The seed words were derived from the words used to describe the themes in the studies. For example, paper 8 described bowel issues using terms such as “diarrhoea,” “losing control of bowel actions,” and “wind”, while paper 12 described this theme using terms such as “nausea,” “constipation,” “gastrointestinal symptoms,” and “poor appetite.” An aggregated list of terms for each theme was used as user-provided seeds to guide the WSTC models and refined during the theme refinement stage.

## 5 Refining the themes

We refined the themes using example comments from a CC PROMs dataset. The dataset is described in Section 7.1. This process helped us to assess the distinctiveness of the themes in real-world data and to refine the themes for PROMs data. We sought input from the domain experts, as explained in the following.

To refine the themes, three domain experts (AD, AWG, RW) with expertise in PROMs were used to improve patient outcomes. These experts were also involved in the collection and original analysis of the CC and PC PROMs data used in this paper. The PC dataset is described in Section 7.1. AWG is a paediatric medical oncologist who uses PROMs to understand the needs of individuals living with and beyond cancer. AD is a cancer epidemiologist whose research focuses on using PROMs data for improving health practice and patient outcomes. RW is a health scientist with research experience in patient-reported outcomes including PROMs comments.

The experts independently annotated 100 comments using the themes from the scoping review, and where applicable, they also provided notes, such as on missing themes. They were provided with the comments, themes, and related seed terms. Inter-annotator agreement was estimated for each theme using Krippendorff’s alpha (α) (52), as the agreement was among the three annotators. Patient comments with very high or low agreement, as well as comments containing issues such as missing themes, were used as discussion prompts. Based on these discussions, a revised list of themes was identified by consolidating similar themes, removing overlapping themes, and adding missing themes.

The experts repeated this process using the revised themes on an additional 200 CC comments to determine the final set of themes. The agreement was calculated (Table 1). When the agreement was at least moderate for all themes, majority voting among the annotators was used to assign a gold standard label to each PROMs comments.

**Table 1:** Agreement scores and theme prevalence among three expert annotators for a sample of CC and PC PROMs comments.

Theme	CC agreement	PC agreement	CC prevalence (*n* = 200)	PC prevalence (*n* = 100)
Cancer pathway and services	0.790	0.838	43% (86)	36% (36)
Comorbidities	0.728	0.864	22% (44)	11% (11)
Daily life	0.608	0.756	16% (31)	16% (16)
Physical function	0.653	0.813	18% (36)	39% (39)
Psychological and emotional function	0.605	0.689	19% (37)	17% (17)
Social life	0.635	0.696	19% (37)	11% (11)
No themes present			10% (19)	5% (5)

The table presents the prevalence of themes in the annotated sample (majority vote labels). A score of 0.61 was considered the suitable threshold for the annotators. Agreement levels are interpreted as follows:0.41–0.60 indicates moderate agreement, 0.61–0.80 indicates substantial agreement, and ≥0.81 is considered “almost perfect.”

To evaluate the generalisability of the themes, independently, the experts annotated 100 PROMs comments from a separate PC dataset. The agreement was calculated, and any issues regarding the annotation were discussed. The themes were appropriate for both datasets. The final themes, definitions, and annotated comments were then used as a framework for annotators to further annotate a sample of the dataset for model evaluation.

## 6 Results of identifying and refining themes

**Figure 2:**
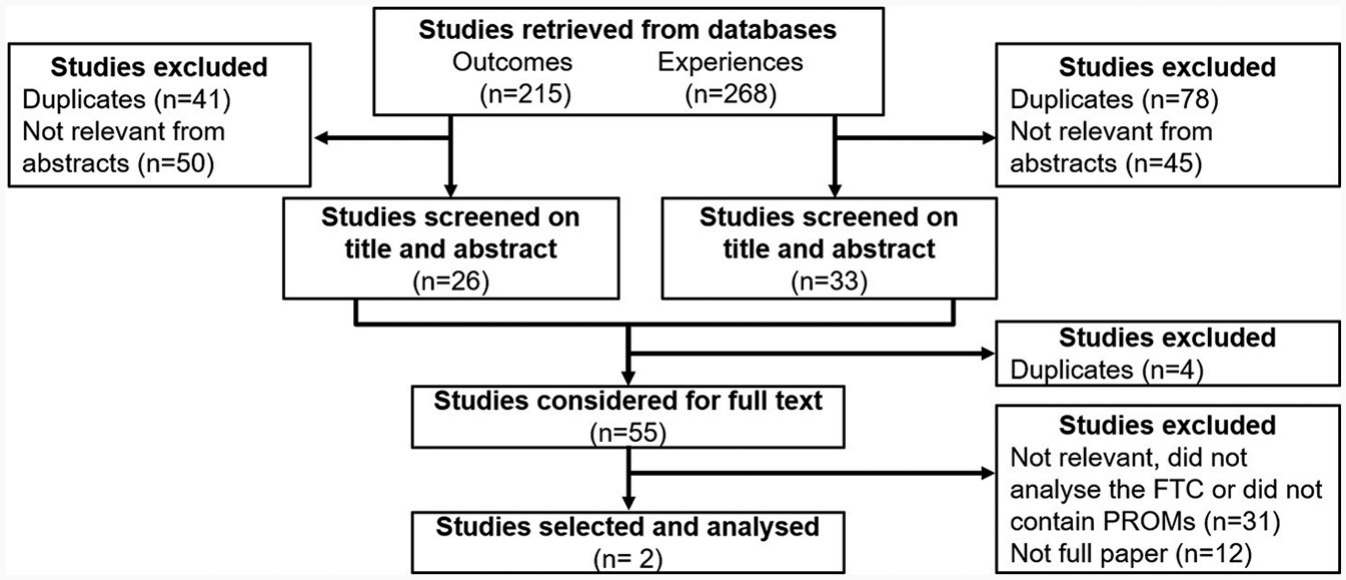
Study selection flowchart. Studies reporting the themes identified in PROMs comments by patients with chronic conditions were searched. From the studies, the reported themes were extracted as reported and grouped based on prevalence and similarity.

Our scoping review identified studies that analysed themes reported by patients with chronic conditions. Figure 2 presents the decision process for study selection. This process includes the results from the search, removal of duplicate citations, study selection, full-text retrieval and additions from reference list searching, and final selection for inclusion in the scoping review.

The database search yielded 215 results related to patient outcomes and 268 results related to patient experience. After removing duplicates and screening based on abstracts, 55 studies were screened for full-text review for inclusion. The final synthesis included 12 records.

The 12 studies explored the responses from patients with 15 different health conditions. These studies included patients with 10 types of cancers (bladder, *n* = 1; breast, *n* = 4; colorectal, *n* = 3; haematological, *n* = 1; leukaemia, *n* = 1; melanoma, *n* = 2; non-Hodgkin’s lymphoma, *n* = 1; prostate, *n* = 3; uterine, *n* = 1; and cancer type not specified, *n* = 3), arthritis (*n* = 2), congestive heart failure (*n* = 1), diabetes (*n* = 1), inflammatory bowel disease (*n* = 1), and pelvic floor surgery illnesses (*n* = 1). The studies were conducted in the USA (*n* = 4), UK (*n* = 4), Australia (*n* = 3), and Canada (*n* = 1). The size of the datasets ranged between 18 and 2,057 responses (mean = 702). The data in these studies were analysed using qualitative methods including grounded theory analysis (*n* = 1), content analysis (*n* = 4), or thematic analysis (*n* = 7).

Table 2 presents the final set of themes after identifying and refining the themes from the scoping review. The final themes were “Cancer Services and Pathways,” “Comorbidities,” “Daily Life,” “Physical Function,” “Psychological and Emotional Function,” and “Social Function.” These themes are broad and high level, with the intention that further analysis would enable characterisation and “zooming in” on the subthemes contained in each theme.

**Table 2:** Final classification of themes along with their subthemes.

Main themes	Subtheme
Cancer Pathway and Services	Cancer pathways
	Health services
	Comorbidities
Comorbidities	Old age and frailty
	Physical activity
Daily Life	Daily life
	Daily activities
	Physical symptoms
	Sex issues
	Sleep
	Weight and appetite
	Pain
Physical Function	Bowel issues
	Memory and concentration
	Mobility
	Sex issues
	Sleep
	Psychological issues
	Body image and identity
Psychological and Emotional Function	Negative feelings
	Positive feelings
	Personal beliefs/spirituality/religiousness/outlook on life
	Financial and employment
Social Function	Social life and relationships
	Support groups and networks

The annotators were provided with these themes alongside theme descriptions.

As the PROMs comments could mention multiple themes, each comment could be annotated with up to all of the six themes. Below are two examples of PROMs comments and their corresponding labels:

“Trouble planning to go out as I never know when I urgently need to be near a toilet as I have no control over my bowel.” (**Labels:** Daily Life, Physical function)

“Since my diagnosis I have had considerable pain after I have used the toilet this is so severe I need to take pain relief. This is not relieved unless I take pain relief. This can happen up to 4/5 times a day. This upsets me a great deal. It also stops me socialising.” (**Labels:** Physical Function, Social Function)

The themes were found to be applicable to both PROM comments datasets, although their prevalence and agreement scores varied. The agreement scores for both the CC and PC comments are presented in Table 1. In the PC sample, the experts found fewer comments that contained no themes, with 5% (*n* = 5) of comments containing no theme compared to 10% (*n* = 19) in the CC sample. Notably, across both datasets, some themes, such as “Cancer Pathway and Services” and “Comorbidities,” had a higher agreement score than the other themes, while the “Psychological and Emotional Function” theme had a lower agreement score.

## 7 Classifying themes: keyword-based weakly supervised classification

Using the themes derived from the previous stage (identifying and refining the themes), we aimed to evaluate the extent to which WSTC can be used to label PROMs comments with HRQoL themes. We explored several WSTC methods on two cancer datasets.

### 7.1 Data

Table 3 describes the two cancer PROMs datasets used in this study. The first dataset, Living With and Beyond Bowel Cancer survey data (53), is a CC PROMs dataset comprising responses to a single open-ended question at the end of the survey, with 25% of the respondents providing PROMs comments. The second dataset, Life After Prostate Cancer Diagnosis (54), is a PC PROMs dataset, comprising responses from open-ended questions at the end of each section of the questionnaire and a final generic question at the end of the questionnaire (a total of seven questions). Respondents of the survey could respond to all or none of the questions, and 69% of the respondents (*n* = 21,036) provided at least one PROMs comment.

**Table 3:** Description of datasets, including the description of the size of the two PROMs datasets used in this study.

Dataset	Cancer type	# Docs	Size of the test set	Mean tokens (±SD)	Min tokens	Max tokens
Living With and Beyond Bowel Cancer	Colorectal cancer	5,634	814	43	1	269
Life After Prostate Cancer Diagnosis	Prostate cancer	59,768	1,000	23.4	1	365

SD, standard deviation.

The mean, minimum, and maximum number of tokens in the comments in each dataset are also described.

### 7.2 Keyword-based weakly supervised classification method

We applied five prevalent keyword-based WSTC methods that have previously been evaluated on short text data. These methods were selected to assign, where applicable, more than one HRQoL theme, as comments could contain multiple themes. These models represent a range of approaches to keyword-based WSTC in practice, enabling a comprehensive exploration. We used three topic-modelling-based approaches and two neural network-based approaches.

• **Guided LDA**—Latent Dirichlet allocation (LDA) is a generative statistical model that has prevailed in the literature, often as a baseline, and provides a reliable baseline for WSTC (22). Guided LDA is a modification of the standard LDA model that uses seed words provided by the user as word-topic priors to instantiate the topics.
• **Guided BERTopic**—BERTopic is an embedding-based method that uses pre-trained BERT embeddings and has shown advantages by providing continuous, rather than discrete, topic modelling (55). We used the Guided BERTopic version, which creates dense vector embeddings of the documents using the BERT pre-trained language model. These embeddings are compared to the embeddings for each seeded topic to assign the relevant topics.
  ○ **Anchored CorEx algorithm**—It is a semi-supervised classification method that identifies maximally informative topics through document correlation (21). Topics are “anchored” through provided seed terms, where the model is guided to learn representations that are most relevant to the themes specified through keywords. Instead of using a generative statistical model like GLDA, this approach learns maximally informative topics via an information-theoretic framework.
  ○ **WeSTClass**—It is a neural network-based method that uses a list of seed words to generate pseudo-documents for pre-training. The model is refined in a self-training module on real documents using bootstrapping to predict the labels of the documents. WeSTClass is a state-of-the-art keyword-based WSTC model.
  ○ **X-Class**—It is a neural network-based method that demonstrated state-of-the-art performance for WSTC that uses only one keyword for classification. Expanding provided keywords (surface labels) to a list of seed terms, this method uses BERT embeddings to create pseudo-documents representative of each class and document-class pairs. These pairs are used to train a supervised model.

The parameters used for each these methods were optimised based on the performance of the methods over all range values through a systematic sensitivity analysis. For each model, hyperparameters, such as anchor strength, and label thresholds were varied to assess their impact on the model, which was evaluated based on the performance of the method. The best-performing configuration (code provided in the Supplementary Material) was used for the results reported in this study.

#### 7.2.1 Data pre-processing

For both datasets, we preprocessed the comments for CorEx, GLDA, and WeSTClass. This involved expanding contractions, converting the text to lowercase, removing stopwords, correcting spelling, and tokenising the text. Documents containing one or no words were excluded. The seed terms were processed in the same way. For the CorEx algorithm and GLDA, term frequency–inverse document frequency was used as the word embedding. WeSTClass produced its own embedding as part of the model.

While X-Class and BERTopic typically require raw text as the input, we found that removing stopwords improved performance. Therefore, the text with the stopwords removed was provided as the input instead. For all methods, excluding WeSTClass, unigrams and bigrams were used as seed terms. For WeSTClass, only unigrams were used as seed terms as due to model limitations.

### 7.3 Evaluation

#### 7.3.1 Data annotation

Although labels were not used during the training of WSTC, we produced a labelled sample to assess the model performance. Using domain expert annotations from Section 5, we were able to utilise niche sourcing as described by de Boer et al. (56). This method relies on expert annotations from a small number of experts to guide the annotation of a larger sample by non-experts. These annotators were computing PhD students working in AI and PROMs data analysis. These annotators annotated the same CC PROMs comments as the domain experts in Section 5.

Cohen’s kappa (57) was applied to calculate the agreement between pairs of annotators. Once the agreement based on Cohen’s kappa was moderate (0.4 <α≤ 0.6) or substantial (0.6 <α≤ 0.8), the annotators independently labelled an additional 300 PROMs comments. The PROMs comments, where there was disagreement, were discussed, and the final labels were agreed upon that the final labels had no disagreement. This process was repeated for a subset of PC PROMs comments. Each PROMs comment could contain 0–6 themes. The result of the annotations was a sample of CC and PC PROMs comments labelled with the themes of HRQoL from the scoping review.

#### 7.3.2 Methods evaluation

The methods were trained on the entire dataset and evaluated on a sample (CC *n* = 814, PCa *n* = 1000). To evaluate the performance of WSTC methods, we considered the following metrics:

• Accuracy:
  $$\frac{\text{TP + TN}}{\text{TP + TN + FP + FN}}$$
• Recall
  $$\frac{\text{TP}}{\text{TP + TN}}$$
• Precision
  $$\frac{\text{TP}}{\text{TP + FP}}$$
• F1-score—a harmonic mean of recall and precision
  $$2\times \frac{\text{Precision}\times \text{Recall}}{\text{Precision + Recall}}$$
• Weighted F1—the F1 score weighted by the proportion of each class

$$\sum_{i=1}^{N}w_{i}\times {\text{F}}_{1}{\text{Score}}_{i}$$

Here, TP is the number of true positives, TN is the number of true negatives, FP is the number of false positives, and FN is the number of false negatives. We also carried out a qualitative analysis of keywords extracted from the methods and labelled comments.

#### 7.3.3 Human understandable model interpretation

We extracted the top keywords for each method to gain a human-interpretable insight, or “explanation,” of their mechanisms. The domain experts, as described in Section 5, were provided with 15 keywords per theme from each method. They independently reviewed the extracted keywords and identified those that were relevant to the theme but were not included in the initial seed terms. The domain experts were able to provide observations about the methods and keywords. The identified keywords were aggregated and discussed during a session with all the domain experts.

For CorEx, GLDA, and BERTopic, the keywords were taken from the term–topic matrix acquired during training to provide topic representation. For WeSTClass, the keywords were taken from the words used during pseudo-document generation and were an expansion of the seed terms. Finally, for XClass, the keywords were extracted during the class-oriented document alignment phase. These keywords from XClass are the words with the greatest similarity to the seed words in the vocabulary of the corpus.

## 8 Results of classifying themes and evaluation

We present the performance results of the five methods across both datasets. We explored the quantitative performance of the methods and the interpretability of the methods with respect to non-technical domain experts.

### 8.1 Performance metrics

The accuracy of the methods for multi-class classification across both datasets is presented in Table 4, in which we see variations between methods and datasets. CorEx outperformed the other methods in both datasets (CC = 0.566, PC = 0.607), while BERTopic and XClass exhibited the lowest weighted F1 score for CC (0.331) and PC (0.280).

**Table 4:** Weighted F1 scores of weakly supervised text classification methods on both datasets.

Data	Method				
	BERTopic	CorEx	GLDA	WeSTClass	X-Class
CC	0.331	**0.566**	0.486	0.447	0.418
PC	0.309	**0.607**	0.316	0.569	0.280
Average across datasets	0.320	**0.587**	0.401	0.508	0.349

The best-performing method is in bold.

We further compared the best-performing method for each theme based on the F1 score (Table 5). CorEx provided the highest accuracy for most themes in both datasets. Themes “Comorbidities” and “Psychological and Emotional Function” had the lowest F1 scores for CC (CorEx, 0.511) and PC (CorEx, 0.443), respectively, while themes “Cancer Pathways and Services” (CorEx, 0.676) and “Physical Function” (WeSTClass, 0.690) exhibited the highest F1 scores. Notably, BERTopic underperformed in all themes for CC and in four of the six themes for PC.

**Table 5:** Comparison of the best performance for each theme.

Data	Theme					
	Cancer pathway & services	Comorbidities	Daily life	Physical function	Psychological & Emotional function	Social function
CC	**0.708** (CorEx)	0.756 (XClass)	**0.889** (CorEx)	**0.839** (CorEx)	0.840 (XClass)	**0.900** (CorEx)
PC	0.819 (GLDA)	**0.912** (CorEx)	**0.882** (CorEx)	0.797 (WeSTClass)	0.857 (XClass)	**0.915** (CorEx)

The accuracy and the model that produced that score are shown. The highest accuracy for each dataset is in bold.

**Figure 3:**
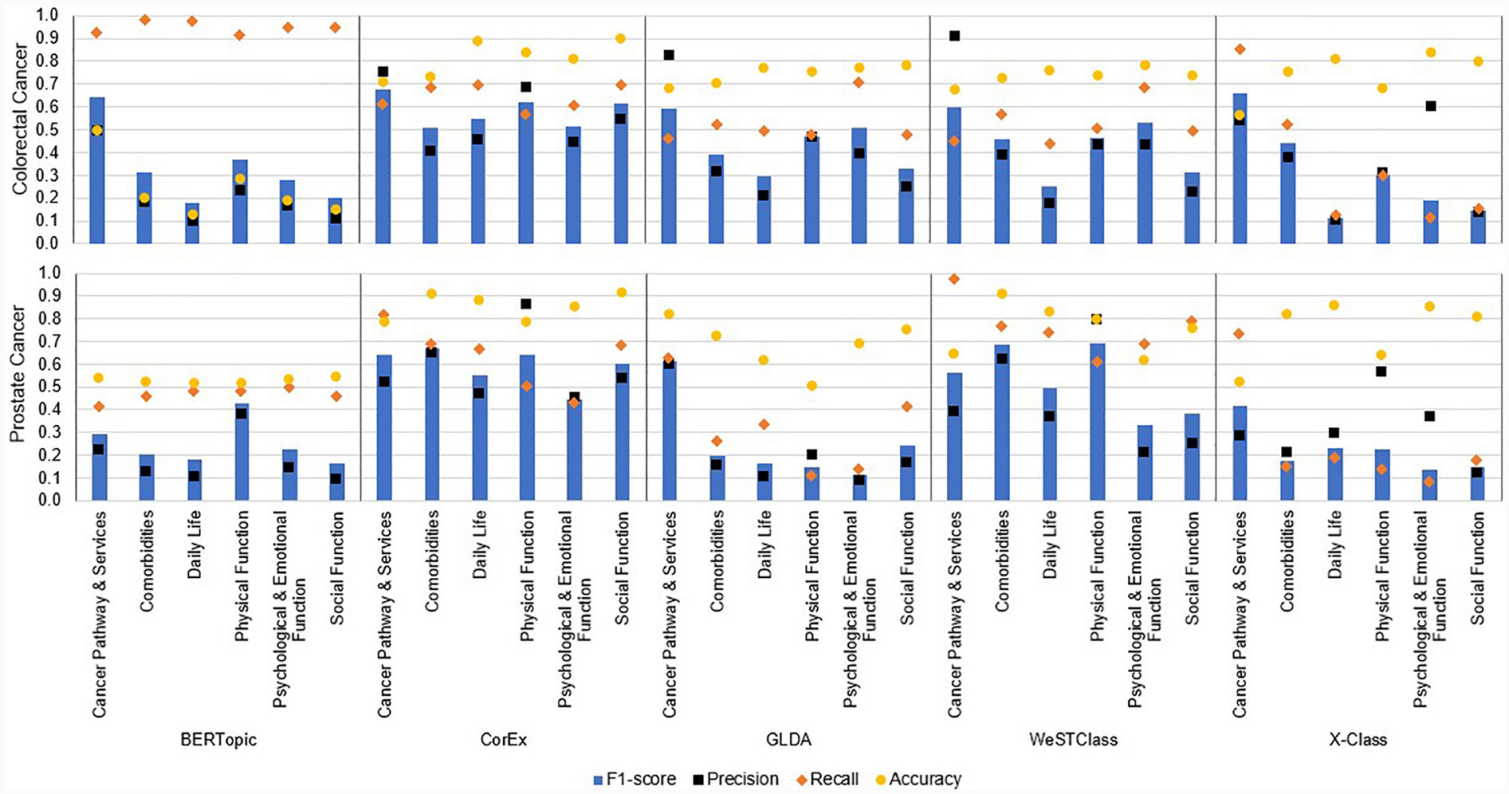
Performance of methods on PC and CC. The F1 score, recall, precision, and accuracy of each method for each theme are presented.

We further explored the performance of the methods by reporting the accuracy, F1 score, recall, and precision for each model by theme (Figure 3). We observed, in many cases, large variations in the performance of the models because of their precision. The “Cancer Pathways and Services” theme was generally well classified by most methods across both datasets, excluding BERTopic on PC PROMs comments (CC: F1 score = 0.59–0.67, Precision = 0.50–0.91, Recall = 0.45–0.92; PC: F1 score = 0.42–0.64, Precision = 0.23–0.58, Recall = 0.52–0.98), while “Daily Life” was typically a poorly classified theme (CC: F1 score = 0.1–0.5, Precision = 0.1–0.4, excluding BERTopic, which was 0.9, Recall = 0.1–0.7; PC: F1 score = 0.07–0.55, Precision = 0.05–0.47, Recall = 0.09–0.74). BERTopic and XClass showed the greatest variation in performance across themes in both datasets, whereas CorEx showed more consistency across themes and datasets.

Precision was the limiting factor for most methods, except XClass, where recall was the limiting factor. There was theme-based variation in precision, as precision was often higher for “Cancer Pathways and Services,” “Physical Function,” and “Comorbidities,” which had higher agreement between the annotators and consisted of quite concrete medical concepts such as “treatment,” “diagnosis,” and “diabetes,” reducing variations due to patient language.

### 8.2 Model interpretability

We reviewed the extracted keywords from each model to evaluate and explore their interpretability for non-technical users. Understanding how the methods classify text is crucial for health research teams to assess the feasibility of using this approach for PROMs comment classification. The extracted keywords were a good indication of what the methods had learned to detect each theme in the PROMs comments and how well they did so compared to quantitative metrics alone. The extracted keywords are presented in Table 6. We found a similar pattern in the extracted keywords for both datasets.

**Table 6:** Keywords extracted from each method (*n* = 15).

Model	Cancer pathway & services	Comorbidities	Daily life	Physical function	Psychological & emotional function	Social function
BERTopic-CC	Filling, form, filling form, completed form, please, process go, writing me, filling information, second form, form out	Treatment, hospital, surgery, cancer, operation, address removed, would, staff, care, bowel	Address removed, name removed, hospital, staff, treatment, address removed hospital, thank, excellent, care, received	Cancer, stoma, surgery, treatment, chemotherapy, bowel, operation, bowel cancer, liver, hospital	Insurance, travel, wife, travel insurance, **positive, attitude**, alone, family, friends, husband	Get life, life keep, life, life thank, it, thank, good alive, see big, thankyou, thanks do
BERTopic-PC	Shock, class, first class, initial shock, first, initial, class **treatment**, class first, **diagnosis**, *shock diagnosis*	Treatment, radiotherapy, cancer, wife, prostate sex, diagnosis, surgery, operation, pain	Weight, **walk, walking**, week, dog, **exercise, diet**, day, golf, weight, gain	Life, future, emotional, normal, worry, anxiety, old, age, fear, positive	None, see, page, impact, nothing, nothing add, add, all, comments, see previous	Decision, surveillance, active, active surveillance, *choice*, consultant, options, made, right, advice
CorEx-CC	**Hospital, nurse, staff, treatment, doctor**, diagnosis, care, *excellent*, screen, **aftercare, surgery**, receive, surgeon, *district (nurse)*, monitoring	Cancer, bowel, bowel cancer, remove, liver, **arthritis**, lung, **stroke**, spread, scan, year, **depression**, ago, tumour, cancer spread	**Diet, exercise, activity, travel, lifestyle, drive, long, term**, hernia, **walk, long term, travel insurance, housework**, *(colostomy) bag*	**Pain**, eat, **bowel movement, weight, diarrhoea, sleep**, foot, **peripheral neuropathy, wind, constipation, tiredness, energy**	**Worry, hope, fear, loss, emotional**, feel, time, come, day, return, know, **faith**, think, thing, happen	**Family, husband, wife, friend, insurance, job, support**, help, financial, child, life, partner, positive, make, *die*
CorEx-PC	**Treatment, radiotherapy, diagnosis, surgery, hospital, doctor, nurse, staff**, diagnose, psa, **operation**, hormone, hormone treatment, **chemotherapy**, cancer	**Arthritis, copd, stroke, heart, dementia, angina, old age, asthma,** knee, **blood pressure, problems, hip, pressure, hypertension**	**Walk, active, activity, travel, drive, exercise, lifestyle, diet,** sexual activity, **vacuum, physical activity, lift, active surveillance,** use	**Sex, pain, sex life, weight, sleep, tiredness, energy, sexual function, weight gain, fatigue, intercourse,** *hot*, impotence, *flushes, hot flushes*	**Worry, loss, depression, emotional, anxiety, confidence, cope, fear, hope, anxious, attitude, depressed, worried, optimistic, relief**	**Wife, family, insurance, partner, relationship, travel insurance, job, financial, friend, friends, social life, support, support family, family friends**
Guided LDA-CC	Care, **treatment, hospital,** receive, *excellent*, **staff, nurse**, *thank*, surgeon, *good*, **doctor**, nhs, diagnosis, team, *support*	Bowel, bowel cancer, scan, remove, liver, surgery, treatment, operation, diagnose, lung, month, year, test, check	Question, problem, answer, year, bowel, prostate, bowel cancer, age, ago, condition, mobility, prostate cancer, heart, relate, old	Operation, bowel, problem, stoma, chemotherapy, day, hernia, time, surgery, month, reversal, cause, foot, leave, control	Support, *nurse*, *information*, need, help, patient, hospital, treatment,*advice, helpful*, surgery, follow, care, *specialist, time*	Life, feel, live, help, positive, think, time, come, day, good, make, **work, family**, look, people
Guided LDA-PC	**Treatment**, would, decision, **surgery**, made, given, told, **diagnosis**, best, choice, consultant, offered, hospital, **radiotherapy**, care	Sex, sexual, lack, erection, life, sex life, incontinence, activity, control, urinary, loss, erectile, sexual activity, none, function	Day, times, get,night, need, tired, toilet, go, sometimes, week, sleep, **walk**, urinate, **walking**, days	Months, psa, prostate, cancer, hormone, weeks, last, tests, removed, blood, diagnosed, years, since, radiotherapy, test	Side, effects, pain, due, problems, side effects, flushes, weight, arthritis, hot flushes, hot, heart, back, prostate, caused	Life, cancer, feel, **wife, family**, prostate, worry, prostate cancer, future, good, old, positive, think, age
WeST Class-CC	**Nurse, doctor, hospital, radiotherapy chemotherapy, surgery, treatment, diagnosis, diagnose, aftercare, referral, screen, monitoring, operation, stoma**	**Angina, heart, diabetes, copd, asthma, ulcer stroke dementia parkinson depression, melanoma, lymphoma, arthritis, old, anxiety**	**Travel, walk, lift, drive, diet, lifestyle, housework, exercise, active, activity, dress, hobby, wash, stairs**	**Nausea, neuropathy, bleeding, cough, cold, fracture, vomit, sleep, weight, appetite, pain, ache, nausea, constipation, diarrhoea**	**Embarrassment, fear, afraid, loss, worry, emotional, gratitude, praise, relief, hope, peace, faith, cope, pray, embarrass**	**Job, employment, family, community, insurance, money, husband, wife, spouse, partner, grandchild, child, social, friend, dependent**
WeST Class-PC	**Doctor, hospital, radiotherapy, chemotherapy, surgery, treatment, diagnosis, diagnose, aftercare, referral, screen, monitoring, operation, stoma, staff**	**Angina, heart, diabetes, copd, asthma,**ulcer, **stroke dementia, Parkinson, depression, melanoma, lymphoma**, arthritis, old, anxiety	**Travel, walk, lift, drive, diet, lifestyle, housework, exercise, active, activity, dress, hobby, wash, stairs**	Months, psa, prostate, cancer, hormone, weeks, last, tests, removed, blood, diagnosed, years, since, radiotherapy, test	**Embarrassment, fear, afraid, loss, worry, emotional, gratitude, praise, relief, hope, peace, faith, pray, embarrass, cope**	Life, cancer, feel,**wife, family**, prostate, worry, prostate cancer, future, good, old, positive, think, age
X- Class-CC	**Treatment**, treatments, therapy, treated, treating, treat, medication, intervention, care, **radiotherapy, chemotherapy**, medicine, **surgery**, clinical, **aftercare**	**Disease**, illness, condition, disorder, syndrome, cancer, infection, failure, cancerous, problem, attack, dysfunction, malignant, symptom, tumour	Lifestyle, life, self, existence, living, live, everyday, *healthy, normally, independent, normality,* lead, **activity**, activities, **hobbies**	Symptoms, signs, complications, *abnormalities*, problems, conditions, issues, difficulties, spots, infections, attacks, effects, reactions, appeared, showing	Psychological, emotional, mental, psychologically, emotionally, mentally, emotions, physically, physical, neurological, depression, feelings, **mood**, traumatic, memory	Social, **socialising**, *public*, **community**, *personal, private, voluntary, group, society,* practical, special, general, *peoples*, people, **friends**
X- Class-PC	**Treatment**, treatments, treated, treating, treat, therapy, medication, intervention, **chemotherapy**, treatable, cure, medicine, procedure,care	**Disease**, illnesses, infections, disease, illness, cancers, *infection, inflammation, attacks*, pneumonia, injuries, burns, sickness, plagues, cancer	Lifestyle, lifestyles, life, style, career, lives, lifes, live, living, environment, personal, lifetime, lived, families, family	Symptoms, signs, complications, conditions, indications, severity, manifested, abnormalities, problems, evident, appears, worsened, apparent, occur, progresses	Psychological, psychologically, emotional, mental, emotionally, mentally, physically, physical, emotions, psychiatric, depression, physiological, emotion, anxiety, mood	Social, **socialise**, socialising, socially, community, society, partytime, sociable, leisure, *public*, supportive, friends, *contact*, conversation, *close*

The terms in bold are also terms in the seed terms provided. The terms that are underlined were agreed upon by the domain experts as relevant to the theme. The terms in italics are words that were included in the themes by one expert with justifications.

Primarily, some methods, such as WeSTClass, adhered strongly to the seed terms provided, while others deviated greatly (BERTopic and X-Class). WeSTClass, unlike the other methods, found few other semantically relevant terms and predominantly contained seed terms (words in bold) in the list of keywords. GLDA captured some relevant keywords but largely included noise and generated themes that were not distinctive, placing keywords such as “arthritis” and “diabetes” in “Psychological and Emotional Function” rather than in “Comorbidities.”

XClass, which uses surface labels, identified relevant keywords except for the “Physical Function” and “Social Function” themes, which contained many irrelevant keywords. In addition, the keywords demonstrated that the surface labels failed to adequately capture the diversity within the themes, as many concepts in the seed terms were not identified in the extracted keywords.

BERTopic produced noisy keywords, deviating from the seed terms and failing to capture them in the top keywords for each theme. For example, “treatment” and “care” were captured in “Daily Life” rather than “Cancer Pathway and Services” and “Physical Function,” respectively. BERTopic fine-tunes a pre-trained BERT model and is anticipated to capture context and nuances better. BERTopic exhibited a good recall, but its performance was likely hindered by insufficient instances to fine-tune the pre-trained model and represent the defined themes sufficiently.

However, the terms extracted from BERTopic highlighted the context of PROMs comments. For example, “friends” extracted from “Psychological and Emotional Function” depicts comments such as

“Optomistic (sic) outlook on life. Supportive family and friends. Healthy diet. Enjoy regular pilates exercises at the gym. Be happy to be alive! Thank you to the NHS for giving me the chance to live.” (**Label**: Psychological & Emotional Function, Daily Life)

CorEx produced a mixture of the original seed terms and additional relevant keywords. “Comorbidities” was the noisiest theme in the CC dataset, with several words relating to CC (primary cancer), such as “bowel cancer” and “scan,” whereas with the PC dataset, the keywords were largely from the provided seed terms. A possible explanation for the quantitative performance of CorEx is that, in addition to expert guidance (the initial seed terms), CorEx also looked at terms derived from the data and thus captured the patients’ context more effectively.

We identified a correlation between the keywords and model performance. Models that captured fewer seed terms and relevant words, such as BERTopic (average F1 = 0.320) and GLDA (average F1 = 0.401), showed lower performance. In contrast, CorEx, which captured primarily seed terms and relevant keywords, achieved the highest performance scores. WestClass, which mainly relied on seed terms, performed well, showing high precision and recall, while XClass, which had relevant words but few seed terms in the keywords, showed lower recall.

The seed terms provided are not extensive and intended to cover the range of concepts within a theme rather than capture all the possible concepts in a theme. Therefore, it was expected that methods that adhered too strongly to the seed terms would perform worse than extrapolating and building upon the seed terms provided. Similarly, the methods that deviated excessively failed to generate themes of relevance. More conservative approaches that prioritise precision are desirable for ensuring that only relevant comments are labelled but this with the risk of producing a very narrow representation of the themes, with a large number of examples missed.

As keyword-based WSTC relies heavily on the quality and relevance of seed terms to the task or dataset, we explored a hybrid way to update the expert-driven seed terms with data-driven term themes (not presented). We included the relevant words highlighted by the experts but found this made little difference to the performance, and in some cases, it improved recall, while in others, it introduced noise.

## 9 Discussion

### 9.1 Key findings

This paper identified the main HRQoL themes reported by patients with chronic conditions and examined the extent to which keyword-based WSTC methods can be used to automatically identify them in unlabelled PROMs comments. We developed a reliable set of patient-reported HRQoL themes to classify PROMs comments and validated them using two PROMs datasets. Investigating the performance of keyword-based WSTC methods quantitatively using performance metrics and qualitatively using the keywords allowed for comparison and interpretation of these methods, which is crucial for healthcare adoption. The WSTC methods in this study employed multi-class labelling, allowing comments to be labelled with multiple themes. Exploring both overall performance and theme-specific performance gave insight into the effectiveness of the methods and highlighted the challenges in the data.

We used the advantage of keyword-based WSTC decrease the need for supervision during training and to reduce the cost of acquiring labelled PROMs comments. Although an effort was invested in deriving the themes to label the PROMs comments (through a scoping review and refinement with domain experts), these themes can be used in any PROMs classification tasks and will allow comparison between PROMs datasets. This is advantageous over unsupervised methods, which identify themes that are data-dependent and do not allow comparison across multiple datasets.

Among the methods explored, CorEx preformed the best (F1 score = 0.587). It appeared that the methods that drew on seed terms provided and inferred additional terms based on the data performed the best overall. We saw characteristic variations between themes. For example, “Daily Life” and “Social Function” are more contextual and subjective themes compared to themes such as Cancer Pathways and Services, which was acknowledged during theme refinement, and may contain more ambiguous concepts that may be more challenging to classify.

Incorporating domain experts into WSTC aided in assessing the approach and its clinical relevance. Their involvement allowed for an approach suitable for classifying unlabelled PROMs comments and useful for end users, i.e., healthcare professionals and researchers (58). The keywords were particularly useful for interpreting results to non-technical audiences for evaluation. This is important for common sense checks of the models that are accessible and understandable for trustworthy adoption.

In addition, relevant keywords demonstrated disparity in experts’ understanding, description of the themes, and how patients discuss the theme in the PROMs comment, which impacted the performance of the methods. For example, “old (age)” was a seed term for comorbidities but was identified in GLDA in “Daily Life.” Whereas from the clinicians’ perspectives, old age is considered a comorbidity, patients often describe aspects of their daily lives that are affected because of old age. Other examples are “thank,” “first-class,” and “excellent” which were captured as “Cancer Pathway and Services.” Although these words are irrelevant to the theme, they indicate how the patients talk about “Cancer Pathway and Services” in the comments. The new keywords picked by the methods can give insight into how patients discuss and provide a clinically valuable context to the themes.

In this study, human evaluation was used to assess the model interpretability and can be employed in future research to compare model performance with human performance. A systematic evaluation involving domain experts, including qualitative researchers who traditionally analyse PROMs comments, can deepen our understanding of how automated metrics align with human preference, such as the trade-off between generalisability and specificity. Human evaluation can also help define the boundaries of themes, ensuring a comprehensive coverage of all the HRQoL topics discussed in the PROMs comments. This evaluation is particularly beneficial for themes with high inter-annotator disagreement.

PROMs comments can contain multiple themes, and in these datasets, several comments contain themes that the annotators considered implied or secondary. This was often the case with more subjective or abstractive themes, such as “Daily Life.” These cases often resulted in disagreement in labels between annotators. In these cases, a certainty or confidence level for each label can be considered, giving a measure of how concretely a theme is present or the degree of inference required by the annotator to determine the presence of the theme (59).

This framework developed to classify PROM comments is generalisable and can be applied to the analysis of other types of patient text. On the other hand, the results of classification—distribution of the themes and extracted keywords—are specific to the datasets used in this study and will be less meaningful to other datasets. The main themes refined from the scoping review and WSTC methods were validated on PC and CC PROMs comments, including those from different survey formats. This validation would suggest generalisability to other cancer PROMs datasets. Generalising the themes to other non-cancer domains would require validation of these datasets. Likewise, the seed terms used in WSTC that represented the HRQoL themes enabling flexibility in the framework, allowing its application to other diseases of interest. This application may require modification of the seed terms to include those more closely related to the themes or diseases investigated.

The topics identified in PROMs and PREMs surveys are often similar, as they are typically limited to the domain of the survey, e.g., specific diseases or the health services they interact with (14). Therefore, patients often report similar themes in PROMs comments, motivating the desire to identify a reliable set of these reported themes. The themes derived were relevant for PROMs comments from cancer patients and were from PROMs with differing formats (single-question vs. multiple-question surveys). While future work would need to examine their generalisability to non-cancer PROMs, this paper only intended to assess their value to these cancer PROMs.

### 9.2 In relation to the existing literature

The growing use of PROMs in both routine care and clinical trials has accelerated the need to readily analyse PROMs comments. PROMs comments are not included in the routine analysis of PROMs due to limited analytical resources, despite their role in elaborating on unmet needs and key influencing factors of health (4, 60, 61). Providing a means to analyse and therefore use free-text comments can help the adherence of patients to complete PROMs (62). Moreover, patients often respond to PROMs to aid future patients (63). Therefore, the insights gained from PROMs can facilitate service evaluation and decision-making focussed on patient needs.

Previous studies have mainly explored the methods selected on single-label documents, potentially affecting their optimisation for multiple labels. This study highlights WSTC performance on short texts within the healthcare domain, where the information is often complex but contextually limited. The challenge of brevity is heightened in weak supervision due to the restricted information present in both the input text and the classification models (64).

This study provides an important evaluation of WSTC performance on short texts within the healthcare domain, where information is complex yet with limited context (64). The methods used in this research were selected because they have been previously applied to short texts (e.g., reviews, comments). However, none of these methods has been evaluated in the healthcare domain, which is a key contribution of the work presented here.

The agreement score demonstrates the challenge of analysing PROMs comments even for domain experts. The challenge is apparent when classifying comments with ambiguity and themes that are typically implied or subtle. Rather than solely being used as an intrinsic limit on expected classification performance (65), the agreement score reveals the challenging and noisy nature of the text and identifies demanding and simple cases. We kept demanding cases from the dataset to maintain real-world scenarios. In future work, it may be of greater value to incorporate disagreement and the variability of expert judgement, such as weak labels and confidence values (67).

### 9.3 Limitations

There are some limitations that we consider for this study. First, the studies selected in the scoping review were conducted in predominantly white, Western countries, often within single-site settings and involving smaller groups of patients. While this can suggest some limitations to the themes that are identified as prevalent, our themes align with the WHOQoL framework, which was validated for cross-cultural suitability, suggesting their representativeness.

Another limitation to consider is that, for both datasets, a subset of comments did not contain any of the six predefined themes. We did not attempt to characterise these comments, and therefore, we did not know the proportion that contained uninformative comments such as “Nope” or novel themes. While some of the models, such as CorEx and BERTopic, can model additional themes beyond the predefined themes, the classification of emerging themes was not explored in this study. The ability to identify novel themes is crucial for understanding evolving patient topics and unmet needs, such as new influences on patients’ HRQoL, including social support from social media (66). Future research could extend the classification of PROMs comments by characterising the comments to identify these novel themes.

In addition, the differing formats of the surveys may have impacted the classification of PROMs comments. The PC PROMs contained multiple questions in the survey, whereas the CC PROMs contained a single open-ended question. This resulted in comments with varying lengths and specificity level. For instance, the CC dataset typically had longer comments, while the PC dataset contained comments that were specific to the question topic, such as their wellbeing, treatment, and impact on their future.

Finally, although we validated the seed terms during the Refining the Themes phase, we did not assess their impact on performance. The domain experts evaluated the seed terms; therefore, we are confident in the domain suitability of the terms. However, Jin et al. (68) showed that the choice of seed terms can influence the performance, potentially adding redundancies or noise to the methods.

PROMs comments show variations in who provides comments and what they report, as such the methods used may be biased towards the common phrases and issues raised by the majority groups, potentially reinforcing existing health disparities (69). Future work would benefit from assessing the impact of under-representation in the PROMs data on model performance considering factors such as sociodemographic, regional and linguistic variations, cultural differences, and specific topics during training or evaluation.

### 9.4 Future work

There are several directions for advancing this work. Future work characterising the themes might prove significant. A deeper insight into HRQoL can be gained by analysing the sentiment of the classified PROMs comments and identifying the subthemes and concepts in the themes. This, in turn, enables more actionable outcomes. Future research can draw more on domain expert knowledge by incorporating approaches such as active learning methods, where algorithms select the most informative data points to be labelled by the domain expert, reducing the number of comments needing manual annotation while achieving a high-performing classifier (70). Such methods can further reduce the demands of the domain experts involved and encourage their involvement in human-in-the-loop-based approaches to improve the reliability and utility of analysis.

In addition, we can look to improve performance by considering hybridising several models and exploiting their strengths. For example, starting with a high-recall method followed by a high-precision model can create a “spam-detection” step before classification. This is useful for datasets with many uninformative comments, such as “nothing to add.” Moreover, combining better embedding techniques with models offering improved guidance towards predefined themes can refine text representation and theme classification.

Significant advancements have been made in the use of large language models (LLMs) for natural language processing tasks (71). Future work can explore their use in weak supervision, including pseudo-labelling comments with ground truths to train classifiers and prompt-based labelling (72, 73).

## 10 Conclusion

Labelling patient-reported free-text data is important to improve the analysis and understanding of HRQoL and its influencing factors from the perspective of patients. Using predefined, known themes to label PROMs comments enables ready and practical analysis of large unlabelled datasets and allows for comparison between methods and datasets. We have successfully identified the usefulness of WSTC for PROMs comments analysis to better understand HRQoL, as it enables the integration of domain knowledge into the analysis process with minimal effort and resource demands, a key factor for future adoption in the routine analysis of PROMs comments.In addition, WSTC offers the opportunity for high-level classification of the PROMs comments.

## Data Availability

The original contributions presented in the study are included in the article/Supplementary Material, further inquiries can be directed to the corresponding author.
