# Supplementary material for: Weakly supervised text classification on free-text comments in patient-reported outcome measures
Source: Front Digit Health. 2025 Apr 30;7:1345360. doi: 10.3389/fdgth.2025.1345360 (PMC12075198; doi:10.3389/fdgth.2025.1345360)
Supplement: Supplementary file 1 [file Datasheet1.pdf]

Supplementary Material

1 SUPPLEMENTARY DATA

Code used for the analysis in the study can be found at `https://github.com/AnnaLinton/WeaklySupervisedPROMs.git`

2 SUPPLEMENTARY TABLES AND FIGURES

Table S1: Themes and associated seed terms. These themes and seed terms were refined with domain experts and used for weakly supervised text classification methods of the PROMs comments.

Themes	Sub-Themes	Seed Terms
Cancer Pathways & Services	Cancer Pathways	radiotherapy, chemotherapy, tamoxifen, surgery, treatment, diagnosis, diagnose, aftercare, referral, screening, monitoring, operation, stoma, medication
	Health services	nurse, doctor, staff, hospital, urgent care, emergency care, community support service
Comorbidities	Comorbidities	Myocardial Infarct, angina, coronary artery disease, congestive heart failure, arrhythmias, hypertension, venous disease, peripheral arterial disease, restrictive lung disease, COPD, chronic bronchitis, emphysema, asthma, hepatic, chronic hepatitis, cirrhosis, stomach/intestine, ulcers, inflammatory bowel disease, IBD, pancreatitis, renal disease, diabetes mellitus, stroke, dementia, paralysis, neuromuscular: MS, multiple sclerosis, Parkinson's, myasthenia Gravis, schizophrenia, major depression, bipolar disorder, AIDS, melanoma, leukaemia, lymphoma, solid tumours, rheumatologic: rheumatoid arthritis, systemic lupus, mixed connective tissue disorder, polymyositis, rheumatic polymyositis, obesity, illicit drug abuse, alcohol abuse, old age, frailty
Daily Life	Activity	travelling limitations, travel, difficulty in walking, physical capacity, usual activities, transportation, restricting activities, activities, activity, avoid activities, lifting heavy objects, taking the stairs, body position, driving

Continued on next page

Themes	Sub-Themes	Seed Terms
	Daily Living	Diet, lifestyle, housework, vacuuming, errands, exercise, daily living, independence
Physical Symptoms	Physical symptoms	hot sweats, jaundice, skin changes, itching, nausea, emesis, dysphagia, peripheral neuropathy, lymphoedema, numbness in fingers and toes, bleeding, physical functioning, migraine, fibromyalgia, injury, breathlessness, shortness of breath, lack of breath, fluid retention, oedema, drowsiness, cough, colds, viral infections, fractures, vomiting, joint stiffness
	Sex issues	sexual dysfunction, sexual function, impotence, sexual difficulties, sex life, sexual positions, intercourse, vaginal dryness, vaginal atrophy, vaginal stenosis, arousal
	Sleep	sleep, disrupted sleep, not sleeping well, interrupted sleep
	Weight and Appetite	weight, appetite, weight gain, eating, weight loss, malnutrition, inability to eat, anorexia
	Pain	pain, ache
	Bowel issues	gastrointestinal symptoms, nausea, constipation, poor appetite, bowel-related concerns, diarrhoea, losing control of bowel actions, wind, gastrointestinal obstruction, gastrointestinal haemorrhage, bowel perforation, bowel complaints, bowel incontinence, urinary incontinence, constipation, bowel movement
	Fatigue/Weakness	fatigue, weakness, tiredness, weakened, energy, stamina, strength
	Memory and concentration	memory, concentration, cognitive problems, diminished memory, distracted, brain fog
	Mobility	balance, poor mobility, mobility problems, limited mobility, physical limitations
Psychological & Emotional Symptoms	Body image and identity	identity, cancer survivor, survivor, body image, perception of body, self-confidence, body appearance, self-esteem, unattractive, ashamed of body, loss of trust in body, sense of control
	Negative feelings	loneliness, negative feelings, mistrust, anger, guilt, emotional distress, embarrassment, fears, afraid, bad thinking, loss, grief, frustration, worry, sadness, fear of recurrence, emotional
	Positive feelings	gratitude, praise, relief, hope, peace

Continued on next page

Themes	Sub-Themes	Seed Terms
	Personal beliefs /spirituality /religiousness	spiritual, faith, meaning of life, optimism, coping, religion, religious, pray
Social Function	Finance and employment	job, redundancy, employment, financial, finance, insurance, money, redundancy
	Social life and relationships	bereavement, husband, wife, spouse, partner, relationship, grandchildren, family, children, child, support from friends, support from family, social life, friends, community, caring responsibilities, dependents, childcare, social self, make plans, socialise, interpersonal, intimacy, isolation, isolated, left on own, alone, intimacy
